# Mandibular Shape Variation, Allometry and Modularity in Adult Mesocephalic Dogs (*Canis lupus familiaris*): Insights into Morphological Integration and Animal Anatomy

**DOI:** 10.3390/ani15223244

**Published:** 2025-11-08

**Authors:** Resef Contreras, Paulo Salinas

**Affiliations:** 1 School of Veterinary Medicine, Faculty of Natural Resources and Veterinary Medicine, Universidad Santo Tomás, Talca 3460000, Chile; 2 Laboratory of Animal & Experimental Morphology, Institute of Biology, Faculty of Sciences, Pontificia Universidad Católica de Valparaíso, Valparaíso 2374631, Chile

**Keywords:** *Canis lupus familiaris*, geometric morphometrics, mandibular modularity, veterinary anatomy, allometry

## Abstract

**Simple Summary:**

Understanding how the dog’s mandible varies in shape is important for veterinary anatomy, since this bone plays a key role in chewing, bite strength, and dental alignment. In this study, we analyzed the shape of 168 mandibles from adult mesocephalic dogs—those with balanced head proportions—using geometric morphometrics, a method that captures subtle shape differences through digital landmarks. The results showed two main mandibular configurations that overlapped widely but differed in the height of the coronoid process and the thickness of the mandibular ramus. Shape variation was only slightly related to size, meaning that larger mandibules did not necessarily differ in form. The mandible also showed a modular structure, composed of regions that vary partly independently, such as the ramus and angular process. These findings help explain how the dog’s mandible can be both functionally stable and flexible, adapting to different breeds and feeding behaviors. This approach improves anatomical teaching, supports diagnosis of mandibular asymmetries, and provides a reproducible method for veterinary, archaeological, and evolutionary studies.

**Abstract:**

The mandible of domestic dogs represents a key structure in veterinary anatomy. This study tested the hypothesis that mandibular shape variation in adult mesocephalic dogs follows a non-random modular pattern with limited allometric influence. A total of 168 dry mandibles from academic osteological collections were analyzed using geometric morphometrics. Four anatomical landmarks and two curves of sliding semilandmarks were digitized and processed through Generalized Procrustes Analysis. Principal component analysis revealed that 62.7% of total variance was concentrated in the first two axes, associated with the coronoid height, ramus robustness, and curvature of the mandibular body. Cluster and Canonical Variate Analyses identified two overlapping but statistically distinct configurations, reflecting the intrinsic morphological diversity of mesocephalic dogs. Procrustes regression confirmed a significant yet low allometric effect (2.34%), while modularity tests based on RV coefficients supported a structured organization involving the ramus, coronoid, and angular processes (*processus angularis mandibulae*) as relatively independent modules. These results indicate that mandibular shape variation is hierarchically organized rather than random, highlighting the coexistence of integration and modular independence within the masticatory apparatus. Beyond its morphometric contribution, this study provides a reproducible anatomical baseline for veterinary and comparative research, facilitating future analyses of sexual dimorphism, functional adaptation, and surgical applications.

## 1. Introduction

Mandibular morphology in dogs (*Canis lupus familiaris*) represents a critical component for understanding phenotypic variability influenced by evolutionary, selective, and functional factors, particularly in contexts where biographical information, such as sex, is unavailable. Previous studies have identified sexual dimorphism in cranial structures of canids, particularly in the basioccipital portion of the skull, where significant differences between males and females have been observed in the elevation and width of triangular areas and pharyngeal tubercles [1,2,3,4]. Similarly, morphometric analyses of the mandibulodental complex in wild and domestic canids have revealed dimorphic patterns, with males showing greater consistency in measurements relative to cranial size, whereas females exhibit a mosaic pattern, suggesting differential influences on functional integration [5]. These findings underscore the relevance of probing latent axes of phenotypic variation in dry mandibles lacking biographical records, while also recognizing that, in domestic dogs, apparent sex-related patterns can be confounded by breed-related heterogeneity and developmental allometry [6].

Geometric morphometrics (GM) has been established as a robust framework for the exploratory and quantitative analysis of cranial and mandibular form in canids, enabling precise evaluation of multivariate shape and the identification of covarying anatomical structures. In both modern and fossil dog mandibles, GM has distinguished forms associated with domestication, highlighting evolutionary differences of the mandible in relation to the skull and documenting covariation across craniofacial units [7]. In dogs subjected to artificial selection for facial shortening, GM has revealed coordinated changes between skull and mandible, with localized effects on functionally critical complexes such as the carnassial, evidencing how brachycephaly reshapes mandibular architecture [8]. Likewise, investigations of ancient dog mandibles have shown unexpected morphological diversity and lower modular covariation compared to modern forms, suggesting that early human selection did not drastically constrain mandibular variability and that organizational patterns have evolved alongside domestication trajectories [9]. Within this comparative landscape, mesocephalic samples provide a pragmatic window onto variation because their intermediate cranial conformation reduces extreme shape departures and can expose subtle but informative morphological gradients. Importantly, positioning domestic dog mandibles within a comparative and evolutionary anatomical framework situates them as a tractable model for examining how developmental constraints, selective pressures, and modular organization shape craniofacial diversity across mammals, including humans.

Two structural features are central to interpreting mandibular shape: allometry and modularity. Allometric scaling, in which size (e.g., centroid size) conditions shape, can structure morphospace and, if unaccounted for, bias inferences about group differences. Studies on endocranial and brain volumes indicate that allometric effects are present in mesocephalic dogs, even when volumetric measures relative to body mass do not diverge markedly from brachycephalic breeds; thus, allometric correction may attenuate—but not eliminate—shape contrasts attributable to other factors [10]. Modularity, in turn, addresses the internal organization of covariation: relatively low and statistically significant between-module covariation (e.g., low RV relative to alternative partitions) indicates that contiguous regions of the mandible can vary semi-independently while remaining integrated as a functional whole [11]. In domestic dogs and wolves, the skull–mandible–dentition complex exhibits consistent patterns of integration, and diversity in dogs is not simply explained by a reduction in modular integration, emphasizing that modular organization and integration co-occur and together constrain and enable morphological change [11]. This framework also accommodates findings that sex effects on cranial shape may not extend uniformly to the mandible once size and age are controlled, underscoring the need to treat putative dimorphism as one component within broader intra-specific variation [6]. Understanding this organization is fundamental for interpreting mandibular biomechanics, planning reconstructive procedures, and improving morphometric teaching models in veterinary anatomy.

Despite these advances, a gap persists regarding the structural organization of mandibular shape variation and its modular patterning in adult mesocephalic dogs when biographical information is unavailable. Osteological collections frequently lack records of sex and breed, and breed diversity itself can inflate or mask signals that might otherwise be attributed to sexual or functional dimorphism [6]. Under these circumstances, geometric morphometrics (GM) is particularly valuable for quantifying the geometry of variation—its principal axes, the contribution of size, and the covariation structure among regions—without presupposing causal attributions. Accordingly, rather than focusing on sexual dimorphism, the present study adopts an explicitly hypothesis-driven and comparative approach aimed at characterizing the structure of morphological variation, the magnitude of allometric effects, and the modular organization of the mandible in adult mesocephalic dogs lacking biographical data. By grounding inference in the geometry of shape and in hypotheses of anatomical contiguity, this approach provides a reproducible baseline for veterinary and comparative anatomy, enabling the analysis of mandibular organization in the absence of demographic metadata. It further contributes to broader discussions on integration, modularity, and evolvability in mammalian craniofacial systems [1,2,3,4,5,6,7,8,9,10,11]. Based on previous evidence of modular organization in the cranio-mandibular complex of canids [9,11,12], we hypothesized that mandibular shape in adult mesocephalic dogs would exhibit (i) a non-random structure of morphological variation concentrated in the coronoid and ramus regions, (ii) a significant but low-magnitude allometric component, and (iii) a modular organization consistent with anatomically contiguous partitions. These hypotheses were tested through two-dimensional geometric morphometrics, evaluating the structure of shape variation, the contribution of size, and the organization of covariation among mandibular regions.

## 2. Materials and Methods

### 2.1. Study Design

This study followed an observational, cross-sectional, and analytical design aimed at characterizing the structure of morphological variation and modular organization in dry mandibles of adult mesocephalic dogs (*Canis lupus familiaris*) lacking biographical records. It was observational because no experimental manipulation was performed; cross-sectional, since all specimens were analyzed within a single temporal frame; and analytical due to the application of multivariate geometric morphometric methods designed to explore patterns of shape variation, size effects, and modular covariation. The study employed a two-dimensional (2D) geometric morphometric approach, a well-established method for evaluating cranial and mandibular morphology in canids [13,14].

### 2.2. Sample and Selection Criteria

A total of 168 dry mandibles were examined, originating from academic osteological collections of Chilean universities (Universidad de Chile, Universidad Santo Tomás, Universidad Católica de Temuco, and Universidad Andrés Bello). All specimens corresponded to adult mesocephalic dogs, defined according to established cranial morphometric indices [15,16,17]. Inclusion criteria required structural integrity, complete disarticulation, and preservation of the anatomical landmarks necessary for digitization. Skeletal maturity was verified through dental chronometry, classifying as adults those specimens with fully erupted permanent dentition—typically achieved after seven months of age [18]. Specimens with fractures, pathological deformations, loss of critical landmarks, or retained deciduous dentition were excluded. All specimen selection and handling were performed by a single operator to minimize observer-related variability.

### 2.3. Digital Image Acquisition

Each mandible was photographed in left lateral view under standardized conditions to minimize orientation bias. The left side was selected to ensure consistency and avoid data duplication, as bilateral asymmetry was not assessed in this study. Images were obtained with an 18-megapixel digital camera (EOS Rebel T3i; Canon Inc., Tokyo, Japan) equipped with a 50 mm fixed focal length lens (Canon EF 50 mm f/1.8; Canon Inc., Tokyo, Japan), mounted on a tripod at a fixed distance of 40 cm perpendicular to the mandibular plane. A neutral, homogeneous background and a metric scale were included in each image. Indirect diffuse illumination was used to reduce shadows and glare, ensuring that observed variation reflected genuine morphological differences. All photographs were calibrated prior to landmark digitization.

### 2.4. Landmark Digitization

Landmark acquisition was performed in TPSDig2 [19]. A mixed configuration of anatomical (Type I) landmarks and sliding semilandmarks was employed to capture both discrete reference points and continuous outlines of the mandible. Four Type I landmarks were defined: (1) *coronion* (tip of the coronoid process (*Processus coronoideus mandibulae*)), (2) *condylion* (caudal-most point on the condylar process), (3) *gonion* (caudoventral point of the mandibular angle), and (4) *infradentale* (midline point between lower central incisors). Two curves of semilandmarks were subsequently digitized: one along the mandibular outline (73 points) connecting gonion and infradentale, and another along the masseteric fossa (*Fossa masseterica*; 13 points) connecting gonion and coronion. Semilandmarks were evenly spaced and allowed to slide along their tangent directions to minimize Procrustes distance among specimens, optimizing geometric correspondence [20,21]. This configuration ensured anatomical anchoring while capturing local curvature and spatial continuity along functionally relevant regions such as the ramus and the alveolar arch. All digitization was performed by a single trained operator following a standardized protocol to minimize intra-observer error.

### 2.5. Morphometric Processing and Statistical Analyses

**Figure 1:**
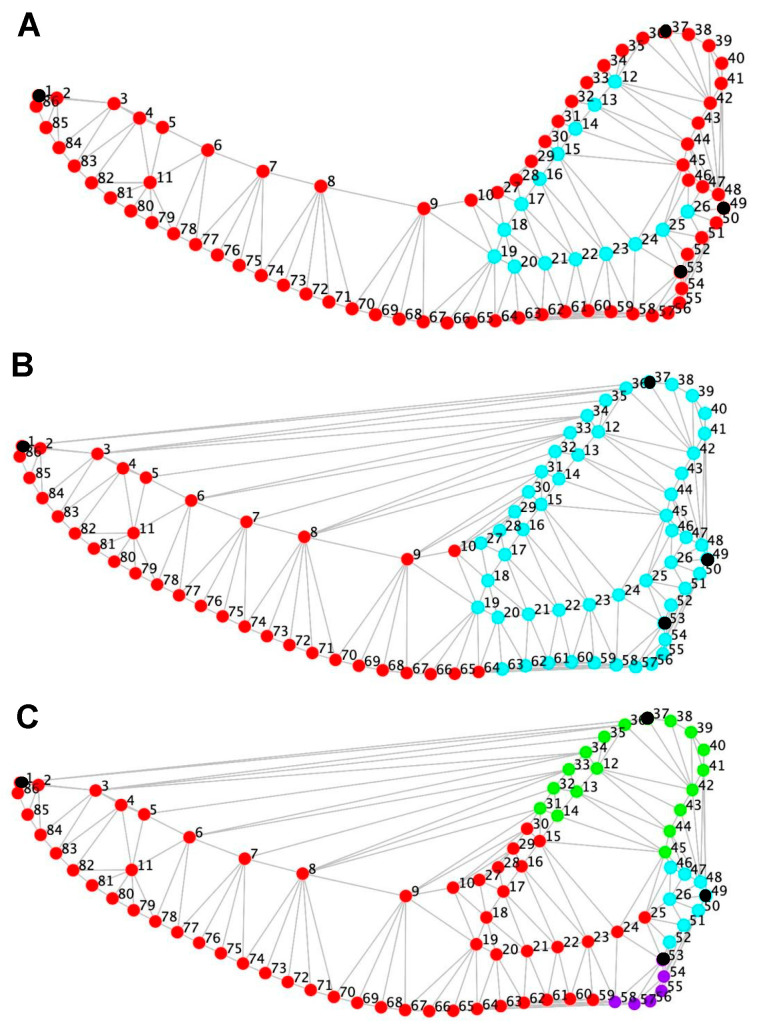
Anatomical configuration and modular partition hypotheses used in the geometric morphometric analysis of adult mesocephalic dog mandibles (*Canis lupus familiaris*). Each configuration shows the spatial distribution of 86 digitized points, including four Type I anatomical landmarks (black nodes)—(1) *coronion*, (2) *condylion*, (3) *gonion*, and (4) *infradentale*—and two curves of sliding semilandmarks describing the mandibular outline and the masseteric fossa. Semilandmarks were evenly spaced and subsequently slid along their tangent directions to minimize Procrustes distance, ensuring geometric correspondence among specimens. Panels (**A**–**C**) illustrate the three modular hypotheses tested in the study, all constrained to contiguous anatomical partitions: (**A**) Partition between the masseteric fossa (cyan) and the mandibular outline (red), capturing potential independence between the insertion area of the masseter muscle and the general contour of the mandible. (**B**) Partition between the mandibular ramus (cyan) and the mandibular body (red), corresponding to functionally distinct regions related to mastication and dental support. (**C**) Four-module hypothesis separating the coronoid process (green), mandibular tubercle (cyan), angular process (violet), and the remainder of the mandible (red), each representing a discrete bony eminence with distinct muscular or articular function. These partition models were evaluated using the RV coefficient as a measure of inter-module covariation, comparing observed RV values against distributions from random contiguous partitions (10,000 permutations). Lower observed RV values were interpreted as evidence of stronger modular organization (Klingenberg, 2009 [22]).

Each analytical procedure was designed to test specific hypotheses regarding shape structure (PCA and clustering), size–shape relationships (Procrustes regression), and covariation patterns among anatomical regions (modularity tests based on RV coefficients). Raw coordinate data were processed in MorphoJ [13]. Generalized Procrustes Analysis (GPA) was applied to remove non-shape variation (position, orientation, and scale) and to compute centroid size for each specimen [14,21]. To describe morphological variation, Principal Component Analysis (PCA) was conducted to identify major axes of shape variation and to quantify the proportion of explained variance. Subsequent cluster analysis (K-means) was applied to PCA scores to explore latent group structure within morphospace, implemented in RStudio (v 4.2.0) using the packages stats, cluster, and factoextra. The number of clusters was initially set to K = 2 for exploratory purposes, and internal consistency was assessed via silhouette coefficients and within-cluster variance. Canonical variate analysis (CVA) was performed in MorphoJ to visualize and maximize group differentiation, with a statistical significance of Mahalanobis and Procrustes distances evaluated by 10,000 random permutations. To evaluate the effect of size on shape, a Procrustes regression of shape coordinates on log-transformed centroid size was computed in R using the procD.lm() function (package geomorph v 4.0), with 10,000 permutations to assess significance. Morphological modularity was evaluated using the RV coefficient [22] as a measure of covariation between predefined anatomical partitions. The observed RV value was compared against distributions generated from all contiguous alternative partitions via permutation (10,000 iterations). Three biologically informed modular hypotheses were tested (Figure 1): (i) masseteric fossa vs. mandibular outline (two-module model), (ii) ramus vs. mandibular body (*Corpus mandibulae*; two-module model), and (iii) bony eminences—coronoid process, mandibular tubercle (*Tuberculum mandibulae*), angular process, and remainder of the mandible—(four-module model). For each model, lower RV values relative to random partitions were interpreted as evidence of reduced inter-module covariation and thus stronger modular organization.

### 2.6. Ethical Considerations

The research involved exclusively skeletal remains from institutional teaching collections, with no interventions involving live animals or experimental procedures. All work complied with Chilean legislation, particularly Law No. 20,380 on Animal Protection and Law No. 21,020 on Responsible Pet Ownership, as well as institutional and international guidelines for the ethical use of biological materials in research. As the study relied solely on pre-existing osteological specimens, formal approval by an animal experimentation ethics committee was not required. Nonetheless, access to and use of the material were authorized by the custodian institutions, and all procedures adhered to established ethical standards for osteological research.

### 2.7. Methodological Limitations

Several methodological considerations qualify the interpretation of results. The absence of biographical data, particularly sex, age, and breed, restricts external validation and limits the causal interpretation of observed morphological clusters. Although adult status was confirmed by complete permanent dentition, postpubertal allometric trajectories may persist and influence features such as the coronoid height or ramus robustness. Dental wear was not systematically quantified, potentially introducing minor variation related to age. From a technical perspective, although the landmark configuration included both Type I anatomical points and sliding semilandmarks, the analysis was conducted in two dimensions, which may omit depth-related components of mandibular curvature. The standardized photographic setup minimized but did not eliminate potential parallax and perspective distortion. All digitization was performed by a single operator, but no formal Procrustes ANOVA of repeated landmarking was conducted; thus, intra-observer repeatability could not be statistically estimated. Finally, while the use of exploratory clustering (K-means) and CVA allowed the detection of latent morphological structures, the non-independence of these analyses entails analytical circularity and therefore precludes confirmatory inference. These caveats emphasize the exploratory nature of the present work and outline directions for future studies incorporating three-dimensional datasets, balanced sexed samples, and formal repeatability assessments.

## 3. Results

### 3.1. Principal Component Analysis (PCA)

**Figure 2:**
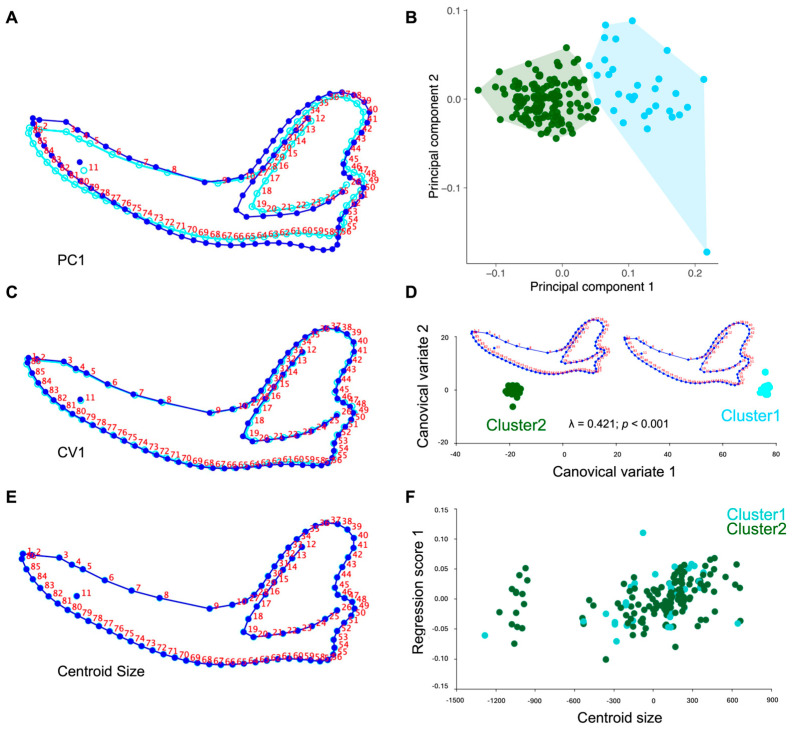
Geometric morphometric analysis of adult mesocephalic dog mandibles (*Canis lupus familiaris*). Shape deformation associated with the first principal component (PC1) explained the largest proportion of total shape variance (**A**). The scatterplot of individual specimens in the morphometric space defined by PC1 and PC2 shows two clusters (Cluster 1, light blue; Cluster 2, dark green) identified through K-means clustering (K = 2) (**B**). Shape deformation along the first canonical variate (CV1) describes the main axis of group differentiation (**C**). The distribution of individuals in canonical variate space (CV1–CV2), together with consensus shapes of Cluster 1 and Cluster 2, illustrates group-level morphological differentiation (Wilks’ λ = 0.421; *p* < 0.001, 10,000 permutations) (**D**). Shape variation associated with centroid size is shown as the allometric trajectory obtained from multivariate regression of shape on log-transformed centroid size (**E**), while the Procrustes regression of shape scores on centroid size depicts the relationship between size and mandibular morphology (**F**). In all deformation plots (**A**,**C**,**E**), the blue line represents the mean shape, whereas blue points with red numbers correspond to the original landmark configuration used in the analyses. The light-blue line (cyan) observed in PC1, CV1, and Centroid Size deformation panels represents the shape predicted at the extreme score along each respective axis (i.e., PC1, CV1, or allometric vector), as computed by MorphoJ to illustrate the directional pattern of morphological change relative to the mean configuration. Thin-plate spline deformation grids are shown with magnified displacements for visualization purposes, and landmarks are displayed in their original analytical positions to preserve methodological reproducibility.

The principal component analysis (PCA) applied to the Procrustes coordinates of adult mesocephalic dog mandibles showed that most of the morphological variation was concentrated in the first axes. PC1 accounted for 53.82% of the total variance, followed by PC2 with 8.86%, together explaining 62.68% of the accumulated variance (Figure 2A,B). PC3 contributed an additional 5.78%, and PC4 explained 5.39%, bringing the cumulative variance to 73.85%. Overall, the first five components summarized 78.71% of the total mandibular shape variation. The graphical representation of the morphospace defined by PC1 and PC2 revealed a wide dispersion of specimens without a distinct separation between groups, although partial clustering tendencies were observed. Shape deformations associated with PC1 mainly reflected variation in the height and projection of the coronoid process and condylar process, whereas PC2 captured differences in the contour of the mandibular body and the orientation of the mandibular angle. These axes thus represented the main gradients of mandibular shape variation and constituted the basis for subsequent clustering, canonical variate, and allometric regression analyses. After identification of a potential outlier in the negative extreme of PC2, the corresponding specimen was re-examined. Although mild dental wear and angular curvature were noted, re-analysis excluding this specimen produced no change in the main PCA structure or modularity results, confirming the stability of the dataset (Figure 2B).

### 3.2. Cluster Analysis (K-Means)

The K-means cluster analysis, applied to PCA scores derived from the Procrustes coordinates, identified two latent morphological groupings within the sample of 168 adult mesocephalic dog mandibles. Cluster 1 comprised 33 specimens (19.6%), whereas Cluster 2 included 135 specimens (80.4%), revealing a markedly asymmetric distribution between groups. In the morphospace defined by PC1 and PC2, individuals belonging to Cluster 1 were located primarily toward the positive extremes of PC1, corresponding to relatively robust morphologies characterized by greater coronoid height, increased ramus breadth, and a more prominent condylar projection. In contrast, specimens in Cluster 2 were distributed toward intermediate and negative PC1 values, showing a more gracile morphology with reduced coronoid height and a smoother ventral margin of the mandibular body. The internal consistency of the two-cluster partition was evaluated using validation indices (e.g., silhouette index), which indicated that the clustering structure was statistically stronger than expected by random assignment.

### 3.3. Canonical Variate Analysis (CVA)

The canonical variate analysis (CVA) applied to the full sample of 168 mandibles revealed the presence of statistically distinguishable morphological configurations. The first two canonical axes concentrated most of the discriminant variation: CV1 explained 67.3% and CV2 28.5%, together accounting for 95.8% of total variance. The statistical contrast based on Wilks’ Lambda was highly significant (λ = 0.421; *p* < 0.001), indicating a robust differentiation between clusters. Mahalanobis distances were also significant after 10,000 permutations (*p* < 0.001), confirming the statistical validity of the distinction (Figure 2C,D). The leave-one-out cross-validation yielded 51.2% correct classification (17 of 33 specimens in Cluster 1 and 69 of 135 in Cluster 2), a value only slightly exceeding random expectation. Cluster and discriminant analyses, therefore, suggested two predominant mandibular configurations (morphotypes) supported by the gap statistic (K = 2). However, classification accuracy was moderate (71.4%) and morphospace overlap remained evident, indicating that these morphotypes represent continuous variation rather than discrete groups. Morphologically, shape deformations associated with CV1 were concentrated in the height of the coronoid process and the robustness of the ascending ramus, whereas CV2 described variation in the curvature of the ventral mandibular body and the inclination of the mandibular angle.

### 3.4. Procrustes Regression (Allometry)

The multivariate regression between mandibular shape (Procrustes coordinates) and the logarithm of centroid size revealed a statistically significant allometric effect. The model explained 2.34% of total shape variation (SS_predicted = 0.020; SS_total = 0.857), with significance confirmed after 10,000 permutations (*p* = 0.0008). Although the percentage of explained variance was low, this value lies within the expected range for geometric morphometric studies, where allometric contributions are typically of reduced magnitude. Shape changes along the allometric gradient exhibited a consistent pattern: mandibles with larger centroid size showed proportionally greater height of the coronoid process, increased ramus robustness, and enhanced condylar projection. Conversely, smaller mandibles displayed a more gracile configuration, with reduced coronoid development and a less pronounced mandibular angle (Figure 2E,F).

### 3.5. Modularity Analyses

**Figure 3:**
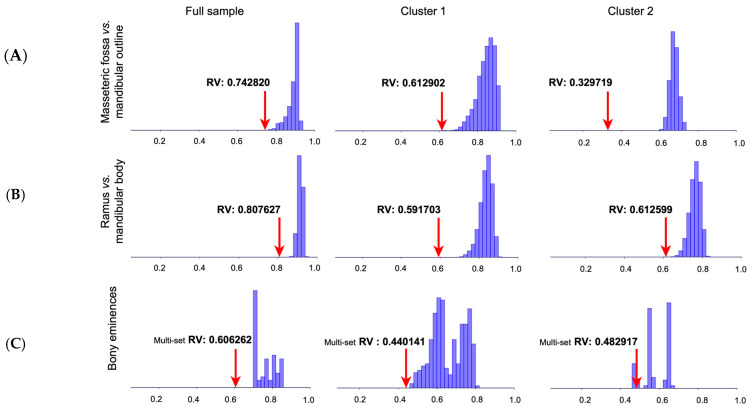
Modularity analyses in adult mesocephalic dog mandibles (*Canis lupus familiaris*). Rows correspond to three anatomical partition hypotheses: (**A**) masseteric fossa vs. mandibular outline; (**B**) ramus vs. mandibular body; and (**C**) bony eminences (coronoid process, mandibular tubercle, angular process, and the remaining mandible). Columns represent results for the full sample (**left**), Cluster 1 (**center**), and Cluster 2 (**right**), as obtained from K-means analysis. Each histogram illustrates the frequency distribution of RV coefficients derived from 10,000 random permutations of contiguous partitions, representing the null distribution of inter-module covariation; the y-axis corresponds to frequency. In the first two rows (**A**,**B**), the x-axis denotes the RV coefficient, whereas in the third row (**C**) the x-axis denotes the multi-set RV coefficient, representing multivariate covariation among ≥3 anatomical subsets. In all panels, the observed RV (or multi-set RV) coefficient for each tested anatomical hypothesis is indicated by a red arrow. Lower observed RV values relative to the simulated distribution indicate reduced covariation among modules, thus supporting stronger modular organization [22]. In the full sample, RV coefficients ranged from 0.606 to 0.808, indicating a generally integrated mandibular structure. Within clusters, lower RV values (e.g., Cluster 2: RV = 0.3297 for the masseteric fossa–outline partition) reflect greater modular differentiation. All observed RV coefficients were statistically significant (*p* < 0.001) when compared with random contiguous partitions.

The modularity analysis between the masseteric fossa and the mandibular outline yielded an RV coefficient of 0.7428 in the complete sample (165 alternative partitions; proportion = 0). In Cluster 1, the value was 0.6129 (720 partitions; proportion = 0), whereas in Cluster 2 it decreased to 0.3297 (59 partitions; proportion = 0). These results indicate reduced covariation—especially in Cluster 2—supporting a relatively higher degree of independence between these regions. The partition contrasting the mandibular ramus (*Ramus mandibulae*) and body produced an RV coefficient of 0.8076 in the complete dataset (13,934 partitions; proportion = 0). In Cluster 1, the value was 0.5917 (117,007 partitions; proportion = 0), and in Cluster 2, 0.6126 (30,757 partitions; proportion = 0). Although all models were statistically significant, the relatively higher RV magnitude in the complete sample suggests stronger integration between these regions overall. The four-module hypothesis—comprising the coronoid process, mandibular tubercle, angular process, and remainder of the mandible—yielded an RV coefficient of 0.6063 in the full dataset (20 partitions; proportion = 0). In Cluster 1, the value decreased to 0.4401 (4 partitions; proportion = 0), and in Cluster 2 to 0.4829 (1 partition; proportion = 0). These lower RV values suggest a clearer modular organization under this anatomical partition, particularly within the groups derived from K-means clustering. Collectively, all modularity hypotheses tested were statistically significant, with the lowest RV coefficients observed in Cluster 2. This pattern suggests that, despite overall integration of the mandibular structure, specific regions—particularly the coronoid and angular processes—exhibit relatively independent patterns of variation (Figure 3).

## 4. Discussion

### 4.1. General

The present study characterized the structure of morphological variation and modular organization in dry mandibles of adult mesocephalic dogs (*Canis lupus familiaris*) lacking biographical records, applying geometric morphometrics through principal component, clustering, canonical variate, allometric regression, and modularity analyses. This approach fills a methodological gap in canid morphology, since most studies have relied on specimens with documented sex or breed [7,11]. Analyzing unrecorded osteological material is particularly relevant for institutional collections, where metadata such as sex, age, or clinical history are often missing. In such contexts, geometric morphometrics provides a quantitative framework to describe morphological variation, identify latent structure, and explore shape geometry [7,21,23,24,25,26].

Rather than inferring sexual dimorphism, the present study used unsexed mandibles to detect morphological differentiation compatible with multiple potential sources of variation, including sex, breed heterogeneity, and functional scaling. The results revealed statistically distinguishable but overlapping morphological clusters, consistent with previous findings in craniofacial structures of dogs and wolves that documented subtle shape differentiation within integrated cranio-mandibular systems [3,27]. The low yet significant allometric effect indicates that mandibular shape variation is only partially size-dependent, while modularity analyses confirmed an organization coherent with reported integration patterns [11].

Together, these findings demonstrate that geometric morphometrics can reveal the internal structure of mandibular variation even in the absence of biographical data, optimizing the scientific use of osteological material in comparative, archaeological, and biomedical research. Importantly, the detected morphological structure should not be interpreted as sexual dimorphism per se but as population-level heterogeneity in mandibular form. Within comparative anatomy, the mesocephalic dog mandible represents a model for understanding general principles of craniofacial variation in mammals. The combination of a weak allometric component, partial group differentiation, and consistent modularity supports that canine mandibular morphology reflects an interplay between integration and independence of functional units—an organization shaped by domestication, developmental constraints, and evolutionary processes [9,10,11,12,21,28,29]. From a veterinary anatomical perspective, characterizing mandibular variability provides a reference for interpreting radiographic and tomographic images, understanding masticatory biomechanics, and recognizing breed-related differences relevant to fracture fixation, dental extractions, and temporomandibular joint evaluation.

### 4.2. Main Axes of Variation in Mandibular Shape

PCA revealed that most of the morphological variation was concentrated in the first two axes, which together explained 62.7% of the total variance (PC1 = 53.82%; PC2 = 8.86%). Shape variation along PC1 reflected changes in the height and projection of the coronoid process, the robustness of the mandibular ramus, and the prominence of the condylar process (*Processus condylaris mandibulae*), whereas PC2 captured differences in the curvature of the ventral mandibular body and the orientation of the mandibular angle. These axes, therefore, represent the principal gradients of mandibular shape variation within the sample and delineate the major dimensions of intra-specific morphological diversity rather than functional or biological groupings. Comparable patterns have been reported in domestic and wild canids, where the coronoid process and mandibular ramus show the greatest morphological flexibility [7]. These structures, together with the mandibular body and angle, respond to selective pressures associated with masticatory function and domestication [9]. Variation in coronoid height and ramus robustness has direct functional implications, as these features determine the lever mechanics and muscle attachment area of the temporalis, influencing bite force and masticatory efficiency. Comparative analyses in canids indicate that domestication relaxed selective constraints on masticatory robustness, leading to reduced coronoid elevation relative to wild taxa such as wolves or foxes [24]. This interpretation aligns with the moderate morphological differentiation observed in our mesocephalic sample. Within the framework of morphological integration, the ramus–body interface has been described as a zone of high covariation, reflecting the balance between local specialization and overall mandibular integration [11]. In this study, the concentration of variance in these anatomical regions supports the finding that the shape gradients identified by PCA parallel those observed across canids generally, indicating intrinsic axes of mandibular flexibility shaped by developmental and selective factors. However, PCA is inherently descriptive and does not imply causal or biological differentiation among individuals. Therefore, the interpretation of morphological structure was refined through K-means clustering and CVA, and the contribution of size was quantified separately via Procrustes regression [7,9,11]. From a veterinary anatomical perspective, the coronoid process, mandibular angle, and body curvature are key landmarks for evaluating occlusal alignment, muscular attachment balance, and mandibular asymmetries. The concentration of morphological variance in these regions reinforces their clinical and functional relevance as indicators of biomechanical stress and masticatory adaptation in dogs.

### 4.3. Differentiation of Morphological Groups

Cluster (K-means) and CVA revealed two latent morphological groupings within the mesocephalic sample, with a markedly asymmetric distribution (≈20% and 80% of specimens, respectively), supported by the Gap Statistic Index (K = 2; Gap = 0.181, SE = 0.012). Cluster 1 comprised mandibles with greater coronoid height, increased ramus breadth, and a more prominent condylar process, whereas Cluster 2 displayed a more gracile configuration, with reduced coronoid elevation and a smoother ventral contour of the mandibular body. CVA indicated a highly significant separation between mean configurations (Wilks’ λ = 0.421; *p* < 0.001), with significant Mahalanobis (94.41) and Procrustes (0.141) distances confirming differentiation between group centroids. However, classification accuracy was moderate (71.4%), and morphospace overlap remained evident, indicating that the separation between clusters is modest and should be interpreted as continuous variation rather than discrete structure (leave-one-out cross-validation yielded 51.2% correct classification; 17/33 in Cluster 1; 69/135 in Cluster 2). The presence of two morphotypes should therefore be viewed as morphometric tendencies within a continuous variation spectrum, likely influenced by allometry and individual variability, consistent with previous findings in canine cranial morphology [1,2]. The identification of overlapping yet distinct mandibular morphotypes provides valuable insight for veterinary morphologists, as these configurations may reflect functional or breed-related adaptations relevant to clinical examination and surgical planning. This morphological variability also holds pedagogical value, exemplifying intraspecific diversity within a standardized skeletal framework. Such a pattern is consistent with the high morphological plasticity of the cranio-mandibular complex in domestic dogs and other canids [1,3,5,11,25,30]. Given that sex and breed were undocumented, the detected clusters are best interpreted as latent structures of shape variation compatible with multiple potential sources—such as functional scaling, masticatory specialization, or breed-related differences—rather than as direct evidence of sexual dimorphism. This interpretation aligns with the broad cranial disparity and pronounced breed stratification of domestic dogs, both of which expand morphometric space and increase shape heterogeneity even within the mesocephalic spectrum [12,31,32,33]. Comparable ordination patterns have been reported in morphogeometric analyses separating canid skulls by cranial type and facial shortening [8,12,34]. Likewise, studies in wild canids reveal subtle sexual dimorphism and measurable craniometric divergence between domestic and wild lineages, supporting that dimorphic signals—when present—are embedded within integrated cranio-mandibular complexes rather than in isolated mandibular traits [35,36,37].

### 4.4. Influence of Size on Mandibular Shape

Procrustes regression revealed a statistically significant but low magnitude allometric effect, explaining 2.34% of total shape variation in relation to centroid size. This value lies within the expected range for geometric morphometric studies, where size contributes modestly to shape variance and functions as a secondary factor relative to other structural or developmental influences [8,10,12]. The small proportion of explained variance indicates that mandibular morphology in adult mesocephalic dogs retains substantial shape variation independent of size, supporting that group differentiation in morphospace is not merely a consequence of allometric scaling. Accordingly, shape differences persisted beyond size-related variation, suggesting that the detected gradients reflect intrinsic developmental or functional variability within the population. This interpretation is consistent with comparative studies in domestic and wild canids, where cranio-mandibular traits maintain identifiable shape components even after controlling for allometric effects [7,9,11]. Thus, while allometry represents an inherent dimension of cranio-mandibular variation, it is not the main driver of morphological differentiation in this sample. Instead, the results point to multi-factorial sources of variation, including functional specialization, developmental integration, and breed-related diversity, which collectively shape mandibular form in mesocephalic dogs. From a veterinary morphological perspective, recognizing these size-independent shape patterns enhances morphometric accuracy for forensic identification, breed standardization, and comparative imaging, reinforcing the clinical and diagnostic value of geometric morphometrics in veterinary anatomy.

### 4.5. Modular Organization of the Mandible

The evaluation of modularity based on three anatomically informed partition hypotheses provided consistent evidence that the mandible is organized into relatively independent structural units. In the model separating the masseteric fossa from the mandibular outline, the RV coefficient was 0.7428 in the complete dataset, decreasing to 0.6129 and 0.3297 in Clusters 1 and 2, respectively. In all cases, no alternative contiguous partition produced RV values equal to or lower than the observed one (proportion = 0), indicating reduced inter-module covariation. Similar results were obtained for the ramus–body partition (RV = 0.8076 overall; 0.5917 and 0.6126 in clusters) and for the four-module model comprising the coronoid process, mandibular tubercle, angular process, and remaining mandible (RV = 0.6062 overall; 0.4401 and 0.4829 in clusters), all with null proportions for alternative partitions.

These findings confirm that modularity arises from the relative comparison of observed versus random contiguous partitions rather than from the absolute RV magnitude. From an anatomical perspective, the identification of semi-independent modules aligns with the distribution of muscular insertions and articular regions recognized in veterinary anatomy. The relative autonomy of the coronoid, condylar, and angular processes corresponds to distinct functional units involved in temporomandibular articulation, masticatory force transmission, and stabilization of occlusal mechanics in dogs. This organization parallels descriptions in domestic dogs and wolves, where the mandible is considered a mosaic of functional units with variable independence, reflecting both evolutionary history and biomechanical specialization [11]. Conceptually, modularity refers to low covariation among modules, while integration denotes strong covariation within them—two properties that coexist in complex morphological systems [13,22,38]. The distinction between the ramus and body modules, together with differentiation of specific bony processes, indicates that mandibular variation is spatially structured and influenced by local developmental and functional constraints. Comparable spatial differentiation has been reported in rodents and other mammals, where selective and mechanical forces promote localized shape variation while maintaining global integration [39]. Lower RV values observed in the clusters relative to the total sample suggest that modularity becomes more evident in morphologically homogeneous subsets, supporting the view that modularity enhances evolvability by permitting localized change without disrupting overall function [28,29]. From a functional standpoint, the partial independence of the coronoid and angular processes is consistent with their muscular and biomechanical roles, which may explain distinct patterns of morphological variation in these regions [6]. Within the broader context of domestic dog diversification, driven by artificial selection and breed formation, the persistence of this modular pattern supports that morphological diversification acted upon preexisting modular frameworks rather than eroding them [8,9,12]. Accordingly, the modularity documented here reinforces the view of the mandible as an evolutionarily flexible and semi-independent component of the craniofacial complex, where localized variation—potentially sex- or breed-related—can accumulate within defined anatomical units while preserving the overall integration of the masticatory system [11,29,39].

### 4.6. Relevance of the Findings

Beyond its morphometric contribution, this study advances veterinary anatomical knowledge by providing quantitative evidence of the modular and integrated organization of the canine mandible. Understanding these structural relationships is fundamental for clinical fields such as veterinary dentistry, maxillofacial surgery, and biomechanics, where anatomical knowledge supports diagnosis and reconstruction. The present evidence reinforces the usefulness of geometric morphometrics for analyzing osteological specimens lacking biographical data, revealing latent structures of mandibular variation in adult mesocephalic dogs. In this context, the combined application of PCA, K-means clustering, and CVA effectively identified statistically significant yet overlapping morphological configurations, while Procrustes regression confirmed a significant but low allometric effect. The limited 51.2% cross-validation accuracy reflects the high morphological overlap and within-group variability typical of domestic dogs, where plasticity and breed diversity broaden mandibular form. Far from reducing the value of the approach, these results highlight its exploratory capacity to characterize shape structure in heterogeneous samples where individual discrimination is inherently limited. Modularity tests consistently showed reduced inter-module covariation, with the lowest RV values corresponding to the ramus, body, and bony processes—the same regions concentrating the main axes of variation. This spatial correspondence between modular partitions and shape gradients demonstrates that GM not only describes form but also provides a hypothesis-driven framework for testing anatomical organization and covariation structure [11,13,39]_._ By integrating allometry and modularity within a single analytical scheme, this study contributes to comparative and evolutionary morphology, positioning the dog mandible as a neontological model for exploring how modular organization and integration sustain craniofacial evolvability while permitting localized variation [7,9,28].

Nevertheless, interpretative caution is warranted. The absence of sex, age, and breed information precludes causal attribution of the observed clusters and limits inference. In osteological and archaeozoological contexts, where such data are typically unavailable, the present procedures may serve as a framework for generating hypotheses and designing classification schemes that can later be validated with sexed or genetically characterized datasets [7,9]. From a veterinary clinical standpoint, knowledge of mandibular variation and modular organization provides an anatomical basis for planning interventions in specific regions, such as the coronoid process or mandibular angle, relevant to temporomandibular joint disorders, malocclusions, and bite alterations [40,41]. Comparatively, the applicability of geometric morphometrics to human craniofacial analyses further supports its methodological robustness, though species-specific validation remains essential [42]. Overall, these findings underscore that geometric morphometrics enables the testing of hypotheses regarding the interplay between allometry, modularity, and morphological integration, contributing to broader discussions on developmental constraint, morphological diversity, and evolutionary flexibility in mammals [7,9,28]. The observed variability in coronoid height and angular configuration likely reflects functional scaling linked to masticatory muscle force transmission. In evolutionary terms, such variation mirrors the reduced mechanical demands associated with domestication and dietary diversification [29].

### 4.7. Limitations

The interpretation of these findings must be tempered by several analytical and methodological limitations. A primary concern is analytical circularity, since group membership was defined through K-means clustering on principal component scores and then assessed for distinctiveness via CVA and DFA. This partial dependence between clustering and validation may inflate Wilks’ Lambda significance, Mahalanobis distances, and cross-classification rates; therefore, the resulting separations should be viewed as exploratory rather than confirmatory evidence of biological differentiation [43,44]. From a sampling perspective, the representativeness of the osteological material may be limited by its provenance and unknown biographical data (sex, age, and breed). Although all specimens corresponded to adult mesocephalic dogs, heterogeneity among institutional collections could introduce unquantified variation related to population structure or husbandry background. A further consideration concerns potential digitization error during landmark acquisition. While all images were processed by a single trained operator to minimize intra-observer variability, no formal Procrustes ANOVA for repeatability was performed. Future work should explicitly quantify this source of measurement error to strengthen reproducibility. Interpretation of mandibular modularity based on RV coefficients must also be cautious: RV values between 0.6 and 0.8 do not necessarily imply weak covariation, and in some partitions, the limited number of contiguous alternatives reduces inferential robustness. Thus, the modularity patterns observed should be interpreted as indicative of relative structural independence, not absolute modular strength. Additionally, the two-dimensional nature of this analysis inevitably omits depth-related components of mandibular curvature and surface topology, particularly in the condylar and masseteric regions. Emerging three-dimensional (3D) geometric morphometric methods using micro-CT, structured-light scanning, or photogrammetry can overcome these constraints, providing anatomically comprehensive models for future validation studies [7,27]. Finally, while the absence of sex, age, and breed metadata limits causal inference, this limitation reflects the reality of most institutional osteological collections. In such cases, geometric morphometrics remains a valid and powerful tool for describing the geometry of variation and testing hypotheses of structural organization, as demonstrated in recent studies on unsexed archaeological and comparative samples [12,27]. Accordingly, this study should be considered an exploratory assessment of mandibular shape structure and modular organization in unsexed mesocephalic dogs, providing a methodological baseline for future confirmatory analyses involving sexed or genetically characterized samples. Integrating morphometric data with clinical imaging or biomechanical testing in future research could enhance the translational value of these findings for veterinary diagnostics and surgical modeling.

### 4.8. Projections

The limitations identified in this study outline a clear roadmap for future research on mandibular variation and modularity in domestic dogs. The foremost priority is the independent validation of the detected morphological clusters using mandibles of known sex, age, and breed, enabling discrimination of variation attributable to biological factors from that derived from collection bias or breed heterogeneity. To mitigate analytical circularity, future studies should implement independent validation frameworks, including train/test partitions, k-fold cross-validation, and permutation-based classification tests, complemented by cluster stability metrics such as the Jaccard Index and Adjusted Rand Index under bootstrap resampling [45,46]. Addressing class imbalance remains critical to ensure robust interpretation of classification performance. Balanced accuracy, F1-scores, and confusion matrices should be reported to avoid misleading global accuracy estimates. Likewise, the number of clusters (K) should be determined empirically through model-based criteria such as the Bayesian Information Criterion (BIC) in Gaussian mixture models, discriminant analysis of principal components (DAPC) with cross-validation, or cluster stability analyses, rather than being imposed a priori. Future work should also extend the assessment of allometric effects, examining within-cluster trajectories, testing their parallelism, and replicating clustering analyses on allometry-free residuals to evaluate robustness. The study of mandibular modularity would benefit from complementary indices such as the covariance ratio (CR), using null models constrained by spatial contiguity, and explicit documentation of landmark configurations for each module. Methodological refinements—particularly combining Type I and II landmarks with semilandmarks, quantifying digitization error via Procrustes ANOVA, and implementing three-dimensional geometric morphometrics—will enhance reproducibility, anatomical interpretability, and biological relevance. Future studies incorporating three-dimensional GM techniques are expected to capture the depth and curvature of the mandibular fossa and condyle, improving the detection of localized covariation and the testing of biomechanical hypotheses. These improvements will also promote the integration of morphometric data with biomechanical and developmental analyses, advancing understanding of how modularity and integration shape mandibular evolution in domestic dogs and other mammals. Integrating geometric morphometrics into veterinary curricula and diagnostic practice could further refine anatomical education, promote evidence-based morphology, and support digital modeling for reconstructive and comparative studies in domestic species.

## 5. Conclusions

The results confirmed the hypotheses proposed for this study, demonstrating that mandibular shape variation in adult mesocephalic dogs (*Canis lupus familiaris*) follows non-random structural axes, exhibits a low but significant allometric effect, and maintains a consistent modular organization. Through a geometric morphometric framework integrating analyses of shape variation, clustering, allometry, and modularity, this work characterized the internal organization of the canine mandible using osteological material lacking biographical data. Principal component and clustering analyses revealed distinguishable yet overlapping morphotypes, mainly differing in the robustness of the mandibular ramus, the height of the coronoid process, and the configuration of the mandibular angle—regions repeatedly identified as morphologically dynamic in canids. Procrustes regression confirmed that size contributes modestly to shape variation, while modularity tests revealed anatomically coherent partitions, particularly between the ramus, body, and bony processes, supporting the hypothesis of a hierarchically organized and integrated mandibular structure. These findings demonstrate that geometric morphometrics provides a hypothesis-driven framework for investigating how allometry and modularity interact to generate morphological diversity, offering more than a descriptive account of form. Within veterinary and comparative anatomy, the mesocephalic dog mandible emerges as a valuable model for understanding how modular architecture underpins both functional stability and evolutionary flexibility in craniofacial systems. Although the absence of sex, age, and breed information limits causal interpretation, the observed morphological patterns align with previously documented sources of variation in domestic and wild canids. Overall, this study provides quantitative and anatomical evidence that modular organization channels morphological diversity under domestication, establishing a methodological and comparative foundation for future confirmatory research incorporating sexed and three-dimensional datasets and expanding the integration of geometric morphometrics into veterinary anatomical and clinical contexts.

## Data Availability

All datasets supporting the findings of this study are publicly available in the Zenodo repository under the record: https://doi.org/10.5281/zenodo.17345072. Two files are included: (1) “PC scores, CovMatrix, mandib_2, Procrustes coordinates.txt”, which contains the Procrustes-aligned landmark coordinates, centroid size values, principal component (PC) scores, and covariance matrices derived from the geometric morphometric analyses; and (2) “graph.abs2.pdf”, which provides the graphical abstract illustrating the methodological workflow and principal morphometric results. Both files are provided under an open-access license and are fully compatible with MorphoJ (v.1.07a) and R (v.4.2.0) environments used in this study.

## Abbreviations

The following abbreviations are used in this manuscript:

ANOVA	Analysis of Variance
BIC	Bayesian Information Criterion
CR	Covariance Ratio
CVA	Canonical Variate Analysis
DFA	Discriminant Function Analysis
DAPC	Discriminant Analysis of Principal Components
GM	Geometric Morphometrics
PCA	Principal Component Analysis
RV	Escoufier’s RV coefficient (measure of covariation between modules)
TPS	Thin Plate Spline
2D	Two-Dimensional
